# Developing a novel treatment for patients with chronic pain and Opioid User Disorder

**DOI:** 10.1186/s13011-022-00464-4

**Published:** 2022-05-07

**Authors:** Amy Wachholtz, Dallas Robinson, Elizabeth Epstein

**Affiliations:** 1 Department of Psychology, University of Colorado Denver, Denver, USA; 2 Department of Psychiatry, University of Massachusetts Medical School, Worcester, USA

**Keywords:** Chronic pain, Opioid use disorder, Treatment development, Comorbidity

## Abstract

**Background:**

It is critical to develop empirically based, community-treatment friendly, psychotherapy interventions to improve treatment for patients with comorbid chronic pain and Opioid Use Disorder. Understanding factors that increase patient adherence and attendance is important, along with strategies targeted to address those issues.

**Methods:**

Based on initial psychophysiology research on adults with OUD and chronic pain, we created an integrated cognitive-behavioral, 12-week outpatient group therapy called STOP (Self-regulation Therapy for Opioid addiction and Pain). In this study, we pilot tested STOP in a Stage 1a feasibility and acceptability study to identify unique treatment needs and factors that increased session attendance, adherence to treatment, and improved outcomes. Fourteen individuals on medication for OUD with co-occurring chronic pain participated.

**Results:**

STOP had high attendance rates (80%; and active patient engagement). Urine toxicology showed no illicit drug use after week 8. Data analysis from pre-intervention to a 3-month follow-up showed significant functional improvement (*F*(1,12) = 45.82;*p* < 0.001) and decreased pain severity levels (*F*(1,12) = 37.62;*p* < 0.01). Participants reported appreciation of the unique tools to counteract physiological activation during a pain flare or craving. Participants also reported benefit from in-session visual aids, applicable pain psychology information, take-home worksheets, tools for relaxation practice, learning to apply the therapy tools.

**Discussion:**

STOP is a 90-min 12-week rolling-entry group therapy based on previous research identifying psychophysiological needs of pain and OUD patients that can be seamlessly incorporated into community addiction treatment clinics.

**Conclusion:**

Preliminary results of STOP are promising with high patient engagement and adherence and significant reductions in drug use and pain.

**Trial registration:**

ClinicalTrials.Gov NCT03363243, Registered Dec 6, 2017.

## Introduction

Opioid addiction and chronic pain are epidemiologically and functionally interrelated. These disorders are often comorbid and require unique therapeutic interventions. Approximately 30–50% of Americans experience non-malignant chronic or repeating pain [1]. Opioid prescribing to treat pain increased from 2007 to 2012 by 7.3% [2, 3]. There was a simultaneous rise in reported cases of abuse of opioid analgesics [4, 5] with estimates of pain-related opioid abuse/addiction up to 50% among individuals with chronic pain [6, 7]. Since 2012, efforts have been made to raise awareness and create safer opioid prescription guidelines, however, the ramifications of opioid overprescribing and the rise in opioid abuse are still prevalent [3].

There is growing recognition of the relationship between opioid use and poor chronic pain management. Eighty percent of patients with opioid addiction entering methadone treatment in the United States also report recent pain,[1, 8] and 37% report chronic pain, described by 65% of people as at least moderately severe [8–10]. Pain is a critical factor in relapse to opioids; individuals with comorbid pain and opioid addiction are 3–5 times more likely to relapse to opioids than those with opioid addiction but no pain [11]. The frequency of concurrent chronic pain and Opioid Use Disorder (OUD) necessitates a better understanding of the psychophysiological links between them, and the need to provide empirically validated integrated treatment options that address both.

Hyperalgesia, which means hypersensitivity to pain, begins to occur in patients within one month of opioid use [11, 12]. Treatment with opioids for pain among those with an opioid addiction history has long been controversial in the academic literature due to concerns that opioids exacerbate hyperalgesia [13], but there is little attention paid to hyperalgesia in treating acute pain among those with a history of OUD [14]. For example, research has shown that individuals with chronic pain have significantly higher odds of reporting craving opioids [15], but the impact of drug craving on pain reactivity has not been widely considered. Further, there is a lack of consensus in the field of addiction medicine about the effect of chronic pain on response to treatment for opioid addiction [16] despite growing evidence that untreated chronic pain creates poorer treatment outcomes for patients with OUD [17].

We have a limited understanding of hyperalgesia related to opioid addiction in the treatment literature, and a limited awareness of the psychological and physiological aspects that inform the pain experience in individuals with a history of opioid addiction. The exacerbation of pain sensitivity among individuals with a history of opioid dependence, combined with the increased risk of relapse to opioids after a pain flare resulting in pain-triggered opioid cravings and abuse, supports the importance of addressing pain in the context of OUD treatment [7].

Sole reliance on medication management to treat this co-morbidity is controversial. In part, this is due to the higher likelihood of Comorbid Opioid use disorder And Pain (COAP) patients to relapse on opioids. It is also influenced by the relationship between elevated pain sensitivity and cravings [14, 18, 19]. The addition of other treatment tools such as cognitive behavioral therapy (CBT) and self-regulation (SR) techniques may be critical treatment components to help COAP patients enter and maintain recovery. One recent study found support for the feasibility and preliminary efficacy of cognitive behavioral therapy in promoting abstinence among COAP patients but did not find significant differences between conditions on pain outcomes [20]. There is still a paucity of empirically validated behavioral treatments for COAP patients, particularly treatments that are grounded in the psychophysiology of both addiction and pain while also providing training to community addiction therapists so that therapists can address comorbid pain. While a systematic review supported the utility of providing training to community addiction therapists on how to manage comorbid chronic pain [21], to this day, legal, ethical, training, and reimbursement issues continue to mean that each issue is often treated separately rather than in a combined treatment approach [22, 23].

A study was previously conducted on psychophysiology to determine the unique psychological and physiological needs of patients with COAP [19, 24, 25] to provide data that can inform both prescribing practices for pain and OUD as well as behavioral interventions for individuals with COAP. This basic science research compared psychophysiological responses to pain and cravings across 120 individuals with chronic pain: 1) on current opioid-agonist medications for OUD (e.g., methadone and buprenorphine), 2) historical treatment with OUD medications but no current opioids, and 3) opioid naïve participants [18, 21, 22].

Participants engaged in a psycho-physiological assessment using a cold-pressor pain task and physiological measures were taken (heart rate, peripheral temperature, galvanic skin response, and frontalis electromyography). Data were also gathered on time to first pain (pain sensitivity), time to disengage from the pain task (pain tolerance), ratings of the pain experienced (pain rating), and level of opioid craving. Finally, participants engaged in a functional assessment to determine the extent to which they were physically limited during specific tasks. This psychophysiological study identified the specific and unique aspects of this patient population to allow for focused interventions in a treatment designed for COAP patients receiving medication assisted treatment. The lessons found below were learned from this psychophysiological needs assessment were critical to the STOP treatment protocol development described in the current study to fill gaps in our knowledge about this patient population.

### Lesson 1

No differences were found between the groups on demographic information, chronic pain baseline level, or pain rating during the standardized cold pressor pain task according to the psycho-physiological assessment measures. COAP participants did not report higher levels of chronic pain nor more acute levels of pain during the cold pressor task compared to the opioid naïve chronic pain participants [19, 24]. Therefore, because COAP participants report similarities to opioid naïve chronic pain participants, some aspects of previously developed and empirically validated pain psychotherapy may be applicable to the current population.

### Lesson 2

COAP participants, regardless of OUD medication treatment status or history experienced pain faster and were able to tolerate it less than the opioid naïve chronic pain participants based on the physiological assessment measures [19]. Therefore, emphasis needs to be placed on pain management self-efficacy techniques that can be easily learned, rapidly implemented, and practiced frequently so that the patient is comfortable using these techniques on demand and independently.

### Lesson 3

Participants currently using opioid agonists (methadone or buprenorphine/naloxone) for OUD treatment had slower physiological recovery (slower return to baseline physiological measures: heart rate, peripheral temperature, galvanic skin response, and frontalis electromyography) subsequent to a pain exposure compared to historical opioid use with current abstinence, or opioid naïve chronic pain participants based on the physiological assessment measures [24]. Therefore, the STOP therapy protocol needs to integrate physiological control techniques to help speed physiological recovery from pain.

### Lesson 4

Even after stopping opioid-agonist treatment for OUD, COAP participants continued to have higher sustained physiological stress compared to opioid naïve chronic pain participants as demonstrated by a consistently lower peripheral temperature measured during the cold-water pressor task. Currently abstinent historical opioid users responded to the pain challenge with more muscle tension than active opioid maintenance and opioid naïve pain participants demonstrated by data collected from the frontalis EMG. The prolonged abstinence group showed better physiological pain tolerance even in the context of greater physiological distress and pain sensitivity, but still faced problems related to their pain psychophysiology despite being abstinent from opioids [24]. Therefore, the STOP therapy protocol needs to educate participants on the variability in duration of pain sensitivity, the unique ways in which their body responds to pain, and why psychophysiological pain response via self-regulation techniques may be useful to reduce the pain experience during and after a pain flare.

### Lesson 5

Among the prolonged abstinence group, as the duration of abstinence grew, individuals in the prolonged abstinence group showed better psychological pain tolerance (though not pain sensitivity). This suggests that individuals with COAP can learn and use techniques that improve psychological tolerance to pain by creating long-term self-efficacy over both chronic pain and opioid use disorder.

### Lesson 6

Participants struggling with COAP also reported significant difficulties with onset, pattern, quantity, and quality of sleep, which can influence pain perception and opioid cravings [25, 26]. Therefore, the STOP therapy protocol should include information on sleep hygiene, quality versus quantity of sleep, and the impact of medications/other substances on sleep quality.

In summary, our initial basic science research showed that those with both current and historical opioid use had increased sensitivity and decreased tolerance to pain compared to an opioid naïve chronic pain group. The development of psychological interventions is essential in patients with both chronic pain and opioid use disorder to reduce physiological pain reactivity, speed pain recovery, and decrease pain distress in the face of prolonged increased pain sensitivity. The six major lessons learned from the psychophysiological study, and unique patient needs, were subsequently addressed during the development of the STOP therapy protocol.

## Methods

Given the high rate of comorbidity and functional interrelatedness, a treatment approach for opioid use disorder and pain that can address both problems simultaneously and utilize self-regulation techniques would be most efficient and potentially more effective that separate treatments. Clinically, these issues are usually addressed sequentially (OUD first, then pain) or do not address the pain at all due to a lack of training by addiction clinicians in pain or by pain clinicians in addiction. There are no empirical data supporting this treatment approach, and it may even hinder treatment for both disorders [27].

In the integrated format of STOP, co-morbid issues are treated simultaneously by the same mental health provider, who has training in both pain and OUD treatment, within a single treatment protocol with the goal of progressing toward resolution of both co-morbidities simultaneously [13]. Using the common factors hypothesis of co-morbidities [28] integrated treatment focuses on mutual goals shared by each co-morbidity treatment. Specific treatments unique to a single issue are then added to the integrated treatment as needed. Based on other integrated treatment studies, this model provides the best opportunity to sustain recovery from OUD in the context of chronic pain [29, 30].

For instance, self-regulation is an effective tool to reduce pain reactivity as well as de-escalate pain-related emotional states and has been called many different things (i.e., relaxation [30], biofeedback [31], autogenic training [32], and imagery [33]). Meta-analyses indicate that self-regulation matches medication in pain reduction [34]. Addiction research on self-regulation is limited, but it has been shown to effectively treat anxiety, cravings, psychopathology, and lead to a reduction in addiction behaviors [33, 35, 36]. Those using self-regulation after drug cue exposure or during treatment were more likely to successfully complete addiction treatment [37, 38]. A small study (N < 10) utilizing CBT and self-regulation showed promising outcomes in addiction treatment with improved opioid use and psychosocial measures [38].

In order to facilitate its eventual adoption, the STOP protocol had to: 1) meet the unique treatment needs of the patients, and 2) meet the needs of community addiction treatment centers who will be using the protocol. Therefore, STOP is based on psychophysiological research to identify and treat the unique needs of the COAP population using an integrated treatment protocol for both OUD and comorbid pain and seeks to increase pain tolerance, lessen drug use, and reduce drug cravings. In addition, STOP was designed to be community treatment friendly with a 12-week outpatient treatment format using rolling entry to allow individuals to join when they are ready and providing therapist training to allow addiction counselors to address both pain and OUD comorbidities while incorporating self-regulation treatment components.

### Model

**Fig. 1:**
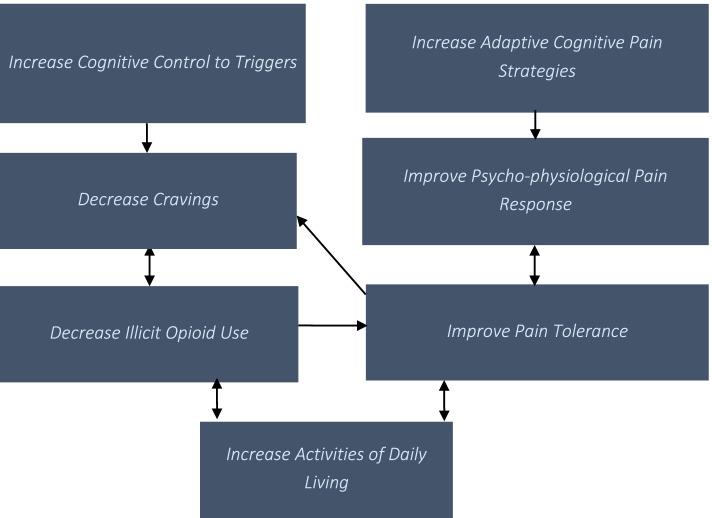
Proposed pathway model of STOP

STOP uses an innovative model of integrated treatment to treat co-morbid OUD and pain which blends CBT and self-regulation treatment (see Fig. 1). In an integrated model, co-morbid issues are treated simultaneously by the same provider within a single treatment protocol. Interactions between co-morbid issues are addressed with the goal of progressing toward resolution and stabilization of both co-morbidities simultaneously [13]. As pain and OUD are intertwined and that a setback in one area may trigger a setback in the other, STOP addresses both areas simultaneously with a psychoeducation plan that teaches both therapists and participants how these two areas can affect each other to create setbacks or build-up of strengths.

#### Format

Traditional research protocols for group therapy primarily use a closed group model [39]. However, 84% of substance abuse psychotherapy treatment uses an open enrollment (rolling entry) format [40] to allow patients to enter treatment quickly and reduce relapse risk or death [41]. The STOP protocol was conceptualized as a rolling entry community-friendly format because the quality of social interaction, commitment to the therapy process, and group alliance does not change based on group membership in rolling entry groups [42]. Rolling entry format is both patient and provider friendly and should allow the final protocol to be seamlessly disseminated and incorporated into medication for opioid use disorder (MOUD) addiction programs, the final successful step to protocol development of dissemination and adoption of the protocol [43].

### Training

Pain management is not typically included in the training for drug and alcohol addiction counselors or master’s level therapists and most doctoral level substance use providers do not have training in pain management as part of their licensure [23]. This lack of adequate training to substantively address pain leaves providers unable to treat patients entering substance abuse treatment with the comorbidity of pain. A critical component of a treatment protocol addressing COAP patients must include a therapist training protocol to address this gap in knowledge with basic pain management education. The current study included field testing of an innovative training protocol for addition therapists naïve to pain management treatment.

Using the information gathered on both patient and community treatment needs, we then developed and piloted a psychotherapy treatment approach to address the unique psychological and physiological needs of this population.

### Initial therapy manual development

The initial STOP manual was developed based on interviews with our community addiction treatment partners about the needs in their treatment population. We selected empirically validated pain treatments and substance use treatments, such as pain education, pain CBT, OUD CBT, relapse prevention, sleep hygiene, and self-regulation therapies, based on previously identified needs from the psycho-physiological studies [19, 25, 26]. Treatments were modified to address the specific needs of the patient population and coalesced into a 90-min, 12-week, rolling entry group therapy treatment called STOP. Sessions used visual images in addition to auditory descriptions for participants who are visual learners, as well as activities to help participants practice the skills from the group. These skills were reinforced with take home worksheets to continue the use of the skills outside of session.

We taught a single relaxation technique that was repeated every session. Participants were asked to practice this relaxation technique daily at home so that they would be extremely familiar with one specific strategy. This method was designed to give participants a known “go-to” and effective tool when they were in the midst of the stress surrounding a drug craving or pain flare. In order to reinforce the use of this relaxation strategy, participants were given Biodots [44] to enhance at home practice. These are small heat sensitive stickers that change color based on the temperature of the participant’s skin (generally a fingertip), similar to “mood rings” that were popular in fashion during the 1970s and 1990s [44]. Biodots provide a cheap form of at home biofeedback during relaxation practice, providing a visual aid for participants to understand the tension and stress in their bodies and to help them reduce it, thereby reducing pain as well. Participants can observe the color of the biodot both at the outset of their relaxation practice, and any changes during their relaxation practice, the Biodot becoming lighter indicating increased heat and blood flow to the fingertip and less muscle tension [44]. This reinforces the physical and mental changes that occur during the relaxation practice taught in session. Biodots are less than 2 cents per dot, have a quick reaction time, and are easy for participants to interpret [45]. Therefore, biodots could be easily incorporated into the therapy process, easily replaced if lost, and do not require any other special equipment to allow participants to engage in effective at-home biofeedback practice.

Each session was group-based and 90 min long. Each session includes: a therapist manual, visual images of key components for each section (for display), participant handouts, and participant homework. Ssessions contained a mixture of didactic, participant practice (e.g., role plays, demonstrations, examples), and interactive problem solving. Visual images and in session skills practice were emphasized to maintain participant engagement, particularly for individuals who may not have strengths in traditional academic skills of verbal and written learning.

The materials were reviewed by experts in the field and revised based on their feedback. We completed an acceptability and beta test of the intervention with a group of five individuals struggling with COAP who were recruited from our community addiction treatment partners. Individuals represented different ages, genders, ethnicities, and phases of treatment. We conducted post-treatment interviews with participants to identify areas that required a final revision. We revised the protocol once again to result in STOP (Self-regulation Therapy for Opioid use and Pain).

### Study therapists

Study therapists were selected for STOP training and providing STOP therapy in the study based on four primary criteria: 1) interest in learning STOP for comorbid pain and OUD, 2) having a master’s degree in a mental health field (psychology, counseling, or social work), 3) experience as a therapist, and 4) willing to work with the study for at least 1 year. Therapists were also required to complete all necessary research ethics trainings.

### Therapist training

Pain management is not a standard component of training for addiction counselors [46]. However, due to the high rate of co-morbidity and the need to develop an addiction treatment community-friendly intervention, a portion of the therapy manual was dedicated to therapist education in pain management to ensure that therapists are able to engage patients with this complex comorbidity and feel comfortable addressing both aspects of comorbid pain and OUD. The STOP therapist training manual provided basic behavioral pain management education to therapists on the topics of basic pain physiology, the interaction between the biology and the psychology of pain, the impact of behavioral pain treatment, and the goal of the pain strategies included in the treatment. The training consisted of a mix of didactic information outlining the rationale and purpose of each of the session topics, applying that information in role plays, and ongoing supervision of therapy sessions through audio recordings. Previous research that trained addiction counselors to treat comorbid disorders indicated that with didactics and supervision, therapists can develop the skills needed to deliver a CBT-based protocol for comorbid addiction and mental health issues [47]. Two master’s level certified addiction counselors received the training for the study. Both therapists were female with 4–5 years of full-time practice.

### Initial training/competency

Therapists received one full day of training on: basic pain physiology, the influence of mood states on pain and cravings, self-regulation and CBT for pain and OUD, influence of sleep on pain, linkages between OUD and pain, and individual skills to increase pain self-efficacy. Formal training contained lectures, demonstrations/modeling, and experiential experiences in small group and 1-on-1 formats. To balance periods of lectures with hands-on training, we provided a lecture introducing a skill or concept, then therapists used this skill in a mock therapy session to allow the trainer to observe their initial grasp of the concept and provide corrections or further skill development as needed. At the end of the didactic training, each therapist had led mock sessions of the key components for STOP. After the completion of the training, including successfully completing the mock sessions under the supervision of a licensed psychologist (AW), the therapist was determined to be competent to provide the therapy.

### Ongoing supervision and training

Study therapists attended weekly group supervision throughout the training and implementation period. Supervision was provided by a licensed clinical psychologist who is a pain and OUD specialist (AW/EE). During these meetings, participant progress (including weekly quantitative and toxicology data), the supervisor’s findings from the audio recordings of previous sessions including adherence and competence, and next week’s session were reviewed.

During weekly supervision, therapists reviewed the next week’s STOP therapy session and provided a practice session presentation during group supervision to allow the supervisor to ensure that therapists fully understood the concepts presented in the session and were prepared to lead the therapy group. Once the therapists displayed basic competency at delivering the manualized treatment sessions as determined by a licensed psychologist (AW/EE), an additional 1-day “booster” training was provided to address more complex issues that may arise in this patient population (e.g., relapse on opioids due to pain flare). Modifications to the therapist training protocol were made based on the clinical experience of the therapists, adherence and competency ratings, subject response, and therapist content knowledge.

All STOP sessions were audio recorded. These recordings were used to assess therapy fidelity using a Therapist Integrity Measure developed for this study. A Therapy Integrity Measure was devised for the current study to assess the therapist’s proficiency and fidelity at delivering the STOP treatment after receiving the STOP training. For each session, 10 specific prescribed interventions or therapist behaviors in the manual were listed (e.g., in session 1, “Therapist was well-prepared with all the materials needed for the session.” “Therapist explained the gate control theory of pain with visual displays.” “Therapist completed the body scan relaxation exercise in a slow, calming manner.”) on a Likert scale of 0 (not at all), 1 (poor skill), 2 (limited skill), 3 (acceptable skill), 4 (considerable skill), 5 (extensive skill). Raters were trained to follow a detailed scoring rubric operationalizing each of the scores 0–5.

This therapy integrity measure allowed us to derive a score for adherence to prescribed interventions in the manual; we tallied dichotomous no or yes delivery score for each intervention in the session (each of 10 items is scored 0 (“no”, not delivered) or 1 (“yes” if rater endorsed 1, 2, 3, 4, or 5) for a total possible adherence score per session of 10, and total possible adherence score of 120 for the entire 12-session protocol. We were also able to derive a session score for quality/level of skill (from poor skill [1] to extensive skill [5] for all of the delivered (i.e., “non 0”) interventions, for a possible mean therapist skill score of 1 to 5 per session or per the entire 12-session protocol.

The first and third authors, both licensed clinical psychologists, independently reviewed audiotapes of STOP therapy sessions for each therapist. Satisfactory fidelity was defined as 90% or greater of delivering the components of the intervention with a competency rating of 4/5 for each item. Once a therapist met both criteria for each session, he or she was identified as proficient for that session. Once full proficiency on STOP was achieved, ongoing ratings of proficiency were made on 30% of randomly selected treatment sessions. Inter-rater reliability for rating the first 12 sessions was 93% for individual items (the discrepant ratings were all within 1 point), and 100% for a dichotomous “pass/fail” decision.

For the groups administered to the 14 pilot participants, the overall adherence score for individual sessions was M = 10, SD = 0, and the overall skill score was M = 4.2, SD = 0.37. Upon completion of the study, we were able to examine overall manual adherence and skill of administration, as well as the broader domains of treatment such as coping with pain and integrative domains such as self-regulation for tolerance of both pain and opioid cravings.

### Participants

Participants were recruited from community addiction treatment centers. Inclusion criteria were English fluency, age 18 to 65 (inclusive), in active treatment for OUD on MOUD, a diagnosed chronic pain condition, and currently stable on their MOUD dose (e.g., not actively titrating up or down). Exclusion criteria were an unstable/untreated psychiatric disorder, recent psychiatric hospitalization (< 3 months ago), or unstable cardiac condition in the past 3 months. Thirty-four participants were initially screened for the current Stage 1a study. Twelve of those screened were ineligible: 10 did not have a chronic pain diagnosis or had a primary substance of abuse other than opioids, and two were not stable on their methadone or buprenorphine dose. Eight individuals were deemed eligible but never appeared for the baseline intake appointments, did not complete informed consent, and did not respond to follow-up contact attempts. There were 14 individuals who were screened, deemed eligible, arrived for their baseline appointment where they provided informed consent for the full study, and completed the baseline assessment.

Fourteen participants (50% female; 50% male) were included in the Stage 1a feasibility and acceptability study of STOP. Participants on average were 43.9 years old (SD = 10.36), with a diagnosed current chronic pain condition (50% had a muscular-skeletal pain source with 50% experiencing mixed neuropathic/muscular skeletal pain, 0% experienced only neuropathic pain). Their mean pain level at baseline was at a 6.1 (SD = 2.90) out of 10 and their mean craving level was 3.9 (SD = 2.52) out of 10. The study sample was 72% Caucasian; 50% on buprenorphine MOUD (M_dose_ = 9.5 mg, SD = 3.0) and 50% on methadone MOUD (M_dose_ = 71.3 mg, SD = 48.55); 50% had some college or higher education level. Ten participants had a diagnosis of Major Depressive Disorder (MDD) and 7 participants had a diagnosis of Generalized Anxiety Disorder (GAD). Two participants were employed, three were seeking work, and nine were on Social Security Disability Insurance. All participants had multiple previous relapses (M = 3.3, SD 2.48) and had been in their current treatment a median of 5 months (including medical withdrawal management). While not required for the study, it should be noted that all the participants had previously dropped out or relapsed from addiction treatment at least once in their history, which suggests that our participant population reflects the typical patient population in a community OUD treatment program.

### Procedure

Participants completed a brief screener to ensure eligibility prior to enrolling into the study. STOP group therapy was provided to participants in their current community addiction treatment center setting, and in collaboration with our community partners, it was offered as a group treatment option in lieu of one of the other mandatory groups they were required to attend to stay in the program and receive methadone or buprenorphine. Throughout the sessions of the study, participants remained stable on a dose of methadone maintenance treatment (MMT) or buprenorphine/naloxone. All procedures were approved by the University of Massachusetts Medical School IRB and the Colorado Multiple IRB (COMIRB) commission.

Participants could enter at any point in the 12 sessions once they had completed medical withdrawal management and medication induction phases of their treatment (See Table 1). There were periodic points of brief review or introduction of the techniques throughout STOP to ensure that everyone had some awareness of the terminology and practices in the protocol regardless of when they entered. If participants missed a session, a brief review of the session would occur by phone or in person prior to the next week’s session.

**Table 1:** STOP session topics for the 12-week group treatment

*Session*	*Topic*
1	Gate control theory of pain, Rationale for pain coping, Addiction/Pain self-efficacy, Role of self-regulation (SR)
2	Self-efficacy beliefs re: controlling pain and drug cravings, Cognitive thought stopping; Practice SR w/biodots
3	Awareness how mood affects pain and cravings, Developing coping thoughts; Practice SR w/biodots
4	Increased somatic awareness related to cravings and pain; Practice SR w/biodots
5	Activity-rest cycling to reduce pain flares; establish personalized cycles; Practice SR w/biodots
6	Problem solving and relapse prevention; Practice SR w/biodots
7	Review/Introduce Skills; Identify progress in treatment of addiction and pain, Pleasant activity; Practice SR w/biodots
8	Establish short- and long-term goals for future behavior change; Practice SR w/biodots
9	Introduction to sleep hygiene and link between sleep and pain; Practice SR w/biodots
10	Explore negative mood, especially anger, as related to pain and drug craving; Practice SR w/biodots
11	Review/Introduce relapse prevention; identify personal/community support resources; Practice SR w/biodots
12	Maintenance plan for positive changes; Practice SR w/biodots

### Baseline, within treatment, and follow-up assessment

In addition to three major assessments during the study: baseline, post-treatment, and 3-month follow-up, participants completed brief weekly assessments of mood, craving, drug use, pain, and a urine toxicology.

Numeric Rating Scale on 0–100 scale was used to rate current and previous week’s pain levels and opioid craving levels [48]. This scale measured week-to-week change, facilitated rapid response by the treatment team if there were any potential negative effects of treatment and allowed participants to track relationship between treatment engagement, home practice, and improvement in pain and craving levels over time. Timeline Follow Back reports were used to collect daily drug use data over the previous week. As a validation of self-report, participants took a 14-panel urine toxicology screen for amphetamine, barbiturates, benzodiazepines, buprenorphine, cocaine, MDMA, methamphetamine, methadone, opiates, oxycodone, PCP, PPX, tricyclic antidepressants, and THC at all assessment points, including weekly [49].

At each of the three major assessment, participants completed urine toxicology, a functional assessment, and an assessment battery. The assessment battery included self-report surveys on craving, distress tolerance, mood, pain levels, pain attitudes, and behaviors, including the Multi-dimensional Pain Inventory [50], a 52-item survey divided into 12 sub-scales. The inventory captures the chronic pain experience across three dimensions including negative life impact, perception of social support, and ability to engage in daily activities.

During the major assessments, participants also did a cold pressor task during which several psychophysiological measures were taken including: heart rate, peripheral temperature (^o^C), frontalis electromyography, and galvanic skin response, in three stages: a 5-min resting baseline, cold pressor task, and a 5-min recovery period. For the cold pressor task, the research asked each participant to place their non-dominant hand up to the wrist in a 2 °C cold water bath. Participants reported when they first experience pain, and remove their hand when, “it becomes too painful.” After the cold pressor task, participants were debriefed using a relaxation exercise and completed a follow-up survey.

At the follow-up assessment points only, participants were administered qualitative interviews related to usage, comfort, and preferences related to the strategies presented in the sessions, the activities, and homework assignments. Participants were compensated for their travel in the form of a one-day bus pass, a one-day medical center parking pass, or a five-dollar gas gift card for each appointment (therapeutic or non-therapeutic) attended, and $20 for each non-therapeutic (e.g., assessment) session at the pre-, post-, and three-month follow-ups.

## Results

### Acceptability

There was an 80% attendance rate of the 12 STOP sessions (Median & Mode: 10 sessions attended) and 100% of the participants who were consented completed the STOP therapy and follow-up assessments. Retention strategies for the study matched those of our community addiction treatment partners. Participants received an automated phone call or email (based on patient preference) 24 h before their appointment, and if they missed a session, they were contacted by the therapist to schedule a telephone make-up session which would be completed prior to the next session. The protocol showed high levels of acceptability compared to the standard community addiction treatment completion rate of 40% [51].

### Feasibility

This therapy protocol was co-developed with community addiction partners to match models used by community addiction treatment providers and enhance dissemination. That development strategy has been effective; during participant recruitment, every site we approached and showed the treatment protocol was enthusiastic about the study and allowed us to recruit participants, as well as requested training for their therapists once the study cycle was completed. Leadership at study sites appreciated the use of CBT techniques, with which many therapists are already familiar, the integration of pain and substance use education in light of the escalating need to treat patients struggling with COAP, and the perceived feasibility of delivery in community treatment settings. The biodots were seen as novel, not prohibitively expensive, and easy to use as a physical biofeedback take home device. The community addiction treatment center leadership shared that many of their patients have unstable cell phone access (e.g., frequent use of pre-paid or “burner” phones), making the use of other biofeedback methods (i.e., smart phone biofeedback apps) untenable for their treatment population.

### Outcome

**Fig. 2:**
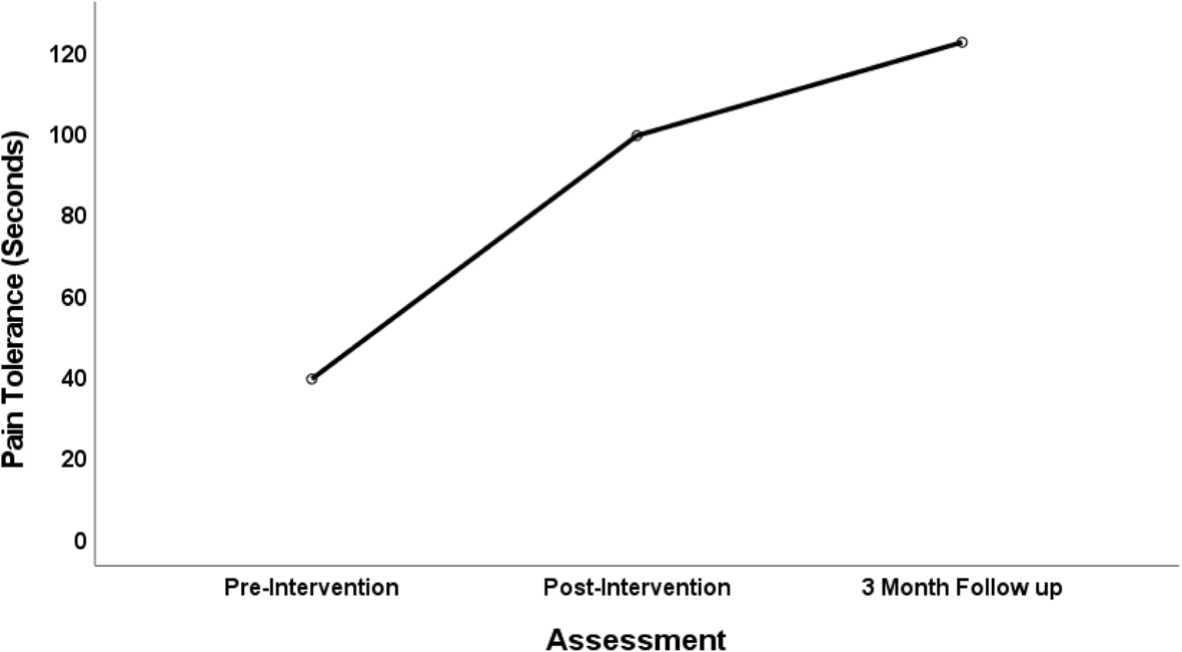
Acute pain tolerance over time

While not powered for efficacy testing, there were promising outcomes in addition to the feasibility and acceptability findings. Participants had no illicit drug (e.g., drugs they were not prescribed as verified by the study team) use after week eight of treatment entry, based on the weekly urine screen results and timeline follow back. Participants also showed significant improvements at the immediate post-intervention assessment that were maintained at the three-month follow-up compared to their baseline. Acute pain tolerance, as measured by seconds in contact with water at 2° Celsius, during the laboratory induced task using a cold-pressor methodology increased significantly from baseline to the post-intervention (12 weeks later; (*F *(1, 12) = 37.62; *p* < 0.001) and was maintained at the three-month follow-up (see Fig. 2). This information allowed us to identify a preliminary Cohen’s D pre-post effect size for acute pain of 0.678 that will allow us to consider when powering future studies. These improvements were also reflected in participants’ physiological reactivity and recovery rates to acute pain.

**Fig. 3:**
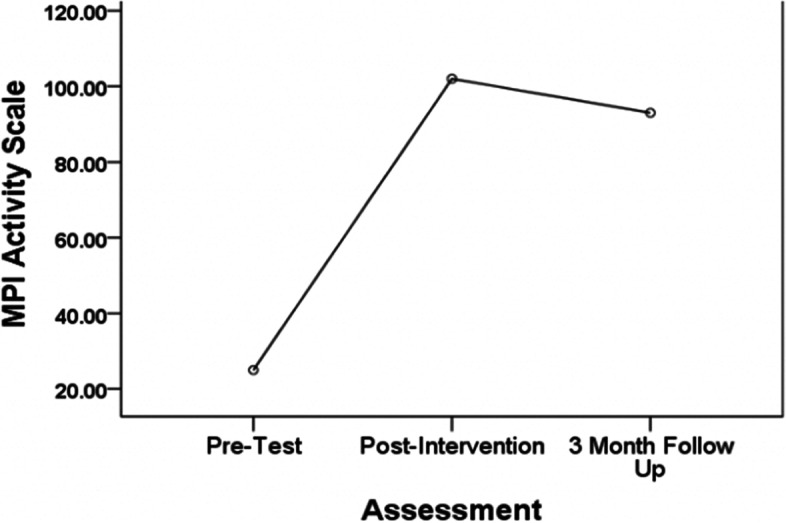
Functional activity over time

Functional activity levels also showed significant improvement from pre- to post- intervention (*F *(1, 12) = 45.82; *p* < 0.001; see Fig. 3). This information allowed us to identify a preliminary Cohen’s D pre-post effect size for acute pain of 0.586 that will allow us to consider when powering future studies. Participants were able to engage in more activities of daily living, household chores, social interaction, and recreational activities over their course of their time in the STOP intervention. These gains were maintained at the three-month follow-up with only a small non-significant drop from the immediate post-STOP evaluation.

### Patient satisfaction with STOP

Participants reported that the intervention was novel (93%), contained information that they had never heard before about pain and addiction (100%), and that the regular practice of a single relaxation skill was useful to them during pain (93%) and craving crises (93%). Whereas 7% reported they would have preferred a variety of relaxation skills to use during pain and craving crises. The Biodot patches were universally reported as being conducive to at home practice of the relaxation technique (100%). Participants stated that the patches helped them to remember to use the technique and that they appreciated the feedback so they could tell if they were doing effective practice. As one participant stated, “[the Biodots] wouldn’t let me do a half-way effort. If I didn’t do it right, it let me know.”

## Discussion

While finding an empirically validated treatment for both opioids and pain is a goal of many US agencies and some early protocols have been assessed, there are no empirically developed protocols to date that integrate both psychophysiology and psychology into a cohesive protocol [52, 53]. The current study sought to fill that gap by examining the psycho-physiological needs of patients with COAP through initial research on the basic science of how psycho-physiological pain response is altered by opioid use [19, 24]. We translated the information into a targeted treatment addressing the psycho-physiological components of both pain and addiction to enhance abstinence sustaining skills to reduce opioid abuse and improve pain functioning. We then piloted STOP, a psychotherapy treatment approach designed to address the unique psychological and physiological needs of this population. The Stage 1a pilot of STOP yielded promising results, improving pain tolerance and reactivity, functional activity, and reducing illicit drug use. These improvements were continued into the three-month follow-up assessment period. The protocol was shown to be feasible and acceptable to participants and community addiction treatment centers, suggesting that once fully validated, the protocol can be easily disseminated, quickly integrated into existing treatment protocols in addiction treatment centers, and used to treat patients struggling with COAP. Future research may also chose to examine STOP in an older population to determine if this protocol meets the unique needs of an older population [54].

One inclusion criterion for this study was that patients be stable on MOUD. Both methadone and buprenorphine have analgesic effects, which could make it difficult to gauge patient’s baseline sensitivity to pain. However, we controlled for the impact of medication by conducting multiple assessments at various time points in the study, and participants did not change their medication regimen during the study. This allowed researchers to assess the changes in the participants’ pain experience after the STOP intervention, without the medication being a confounding factor.

This was a small Stage 1a pilot trial and was not powered for efficacy with an N of 14. However, the protocol is firmly based in addiction/pain physiology as well as addiction/pain psychology and produced promising results. This feasibility study was necessary to develop the therapy protocol and collect preliminary data before implementing it in a larger randomized control trial, consistent with Freedland’s discussion on when a feasibility study is needed [55]. We are currently recruiting for the NIDA R34 Stage 1B pilot randomized control trial (RCT) with community participants with OUD and chronic pain. We anticipate a pilot RCT with half randomized to receive the STOP therapy protocol and the others receiving treatment as usual (TAU) as found in community addiction treatment centers. With this phase in progress and based on the results found in this current study, we hypothesize that those receiving the STOP therapy protocol will have a significantly larger decrease in drug use and pain levels.

## Conclusions

There is clearly a distinct need to develop better COAP treatments that target individuals struggling with COAP and seeking treatment in community addiction treatment centers [55, 56]. These treatments need to be feasible and acceptable to both the patients themselves as well as the community addiction treatment centers who might be providing them or those treatments will fail to be effective or widely disseminated. STOP shows significant promise to meet this serious need in the context of the opioid epidemic as we continue our research utilizing the STOP therapy protocol in community addiction treatment centers.

## Data Availability

The datasets generated and/or analyzed during the current study are not currently publicly available due to the pilot project nature of the study, but the full RCT data will be available once completed from the corresponding author on reasonable request.

## Abbreviations

CBT: Cognitive Behavioral Therapy
COAP: Comorbid Opioid use disorder And Pain
MOUD: Medication for Opioid Use Disorder
MDMA: 3,4-Methyl​enedioxy​methamphetamine, commonly known as “ecstasy” or “molly”
NIDA: National Institute of Drug Abuse
OUD: Opioid Use Disorder
PCP: Phenylcyclohexyl piperidine, commonly known as “angel dust”
PPX: Propoxyphene
RCT: Randomized Controlled Trial
STOP: Self-regulation Therapy for Opioid use disorder and Pain
TAU: Treatment As Usual
THC: Tetrahydrocannabinol, the psychoactive component of cannabis
